# On Hypodermic Injection

**Published:** 1875-11

**Authors:** 


### ON HYPODERMIC INJECTION.

The *Doctor* reports a discussion at the Parisian Medical Society on this subject. M. Martin stated that, even in small doses, an enormous diminution in the force of the pulse and in the arterial tension rapidly took place. The maximum effect was about half an hour after the injection, and the pulse resumed its normal character an hour and a half after. Slight lowering of temperature occurred. M. Martin prefers full doses, particularly in nervous affections, and he does not consider it necessary to inject at the painful spot. If the needle is sharp, the pricks are less painful. The first operations often cause indigestion. This is to be prevented by absolute repose. M. Martin believes the injection to be infallible in all neuralgias, and efficacious against oppressions of all kinds, even those arising from cardiac disease or the access of asthma. He adds that they calm delirium tremens, and arrest obstinate vomiting, even in cases of cancer. At the close of the treatment the doses should be diminished.

M. de Beauvois said that the injections ought never to be administered immediately after meal. He had seen severe precordial anxiety, signs of approaching asphyxia, vomiting, etc., produced by neglect of this precaution. The vomiting relieved the patient from the other alarming symptoms.

M. Duroziez cited the case of a man, eighty-four years of age, suffering from strangulated hernia and incessant vomiting, in whom 3 centigrammes of morphia (nearly half a grain) injected twice caused death. This is a most important fact.

M. Polaillon believes that the hypodermic injection of distilled water by itself will relieve neuralgia and muscular spasms. In such a case we should think the imagination of the patient may have something to do with the cure. This suspicion is strengthened by an observation of M. Peter, who, in a case of heart disease with intercostal neuralgia, injected 0.002 milligramme of atropine. Immediately the patient fell down, pulseless and looking as if dead. Happily, he shortly recovered; and as no symptoms of belladonna poisoning appeared, the cause of the syncope was probably due to the mental impression on the patient. A similar circumstance occurred to M. Krishaber, on injecting a young woman affected with laryngeal pthisis with a small dose of morphia. Some days after, he repeated the injection with distilled water only, and syncope again supervened.

In a patient affected with iataxio, M. Peter injected, for the relief of visceral symptoms, doses of 0.15 to 0.20 cent. of morphia, giving at the same time 0.05 by the stomach. The patient concealed his doses when he was not suffering; one day he took a gramme of morphia by the stomach after 0.20 had been injected. No inconvenience followed this enormous dose. On the other hand may be mentioned a sad fact. A physician troubled with intercostal neuralgia employed injections of morphia in excess. One day a boil appeared at the site of injection; from this erysipelas started, and pleurisy, ending in empyema, supervened. The case terminated fatally.

